# New Data on the Occurrence of Scarabaeoid Beetles (Coleoptera: Scarabaeoidea) in Montenegro

**DOI:** 10.3390/insects13040352

**Published:** 2022-04-01

**Authors:** Adam Byk, Marek Bidas, Tomasz Gazurek, Dawid Marczak, Łukasz Minkina, Sebastian Tylkowski

**Affiliations:** 1 Department of Forest Protection, Institute of Forest Sciences, Warsaw University of Life Sciences—SGGW, Nowoursynowska 159/34, 02-776 Warszawa, Poland; tomasz_gazurek@sggw.edu.pl; 2 Independent Researcher, 25-385 Kielce, Poland; zuk55@o2.pl; 3 Faculty of Engineering and Management, University of Ecology and Management in Warsaw, Olszewska 12, 00-792 Warszawa, Poland; dawid.marczak@wseiz.edu.pl; 4 Independent Researcher, 34-400 Nowy Targ, Poland; klekel@interia.eu; 5 Department of Forest Research, University of Lodz, Branch in Tomaszów Mazowiecki, Konstytucji 3 Maja 65, 97-200 Tomaszów Mazowiecki, Poland; sebastian.tylkowski@uni.lodz.pl; 6 Department of Forest Protection, Forest Protection Division in Krakow, General Directorate of State Forests, Słowackiego 17A, 31-159 Kraków, Poland

**Keywords:** geotrupidae, Trogidae, Lucanidae, Ochodaeidae, Scarabaeidae, biodiversity, distribution, nature conservation, new records, Montenegro

## Abstract

**Simple Summary:**

The Montenegrin insect fauna is still insufficiently researched compared to neighbouring countries. The authors focus on scarabaeoid beetles. The dominant trophic group among scarabaeoid beetles are dung beetles. Dung beetles accelerate the decomposition of faeces, increase soil aeration and reduce parasite (flies and nematodes) numbers. Due to their role in nature, they are sometimes called meadow and forest scavengers. We collected 2130 scarabaeoid beetles belonging to 107 species and 5 families in 34 localities. Collected beetles constitute 54.2% of scarabaeoid beetles recognised so far from Montenegro. Among the fauna collected in this study, 63 species were dung beetles and 16 species were found in Montenegro for the first time. These included *Odonteus armiger*, *Trox sabulosus*, *Ochodaeus integriceps*, *Agrilinus convexus*, *Melinopterus reyi*, *M. sphacelatus*, *Phalacronothus biguttatus*, *Trichonotulus scrofa*, *Psammodius nocturnus*, *Platytomus tibialis*, *Pleurophorus mediterranicus*, *P. pannonicus*, *Rhyssemus berytensis*, *Onthophagus ovatus*, *Rhizotrogus aestivus* and *Chaetopteroplia segetum.* The discovery of so many species in a relatively short time of fieldwork indicates the need to continue and intensify our surveys in the future. Recommendations for the conservation of Montenegrin biodiversity are given.

**Abstract:**

The Montenegrin fauna of the superfamily Scarabaeoidea is not satisfactorily studied. This is evidenced by the small number of species from this superfamily reported from Montenegro, despite the richness of the country’s habitats, especially high-mountain meadows, pastures, lush canyons, riverside, coastal dunes and old forests. Moreover, significant is the greater number of species of scarabaeoid beetles in neighbouring countries. Therefore, we aim to supplement the current information on the distribution of the taxa of the superfamily Scarabaeoidea in the country. The presented scarabaeoid beetles were caught during three expeditions: the first in May/June 2019, the second in May/June 2021, and the third in July 2021. As a result of this study, we have collected 2130 beetles belonging to 107 species and five families of the superfamily Scarabaeoidea: Geotrupidae, Trogidae, Lucanidae, Ochodaeidae and Scarabaeidae. The 28 days of the faunistic study confirmed the occurrence in Montenegro of 54.2% of the scarabaeoid species hitherto known from this country and added 16 new species that had not been previously recorded: *Odonteus armiger* (Scopoli, 1772), *Trox sabulosus* (Linnaeus, 1758), *Ochodaeus integriceps* Semenov, 1891, *Agrilinus convexus* (Erichson, 1848), *Melinopterus reyi* (Reitter, 1892), *M. sphacelatus* (Panzer, 1798), *Phalacronothus biguttatus* (Germar, 1824), *Trichonotulus scrofa* (Fabricius, 1787), *Psammodius nocturnus* Reitter, 1892, *Platytomus tibialis* (Fabricius, 1798), *Pleurophorus mediterranicus* Pittino & Mariani, 1986, *P. pannonicus* Petrovitz, 1961, *Rhyssemus berytensis* Marseul, 1878, *Onthophagus ovatus* (Linnaeus, 1767), *Rhizotrogus aestivus* (Olivier, 1789) and *Chaetopteroplia segetum* (Herbst, 1783). Six species and three subspecies that are typical for the Balkan Peninsula were also found: *Trypocopris alpinus balcanicola* (Mikšić, 1954), *Onthophagus panici* Petrovitz, 1964, *Amphimallon solstitiale simplicissimum* (Müller, 1902), *Omaloplia illyrica* (Baraud, 1965), *Triodontella dalmatica* (Baraud, 1962), *Chaetopteroplia segetum straminea* (Brullé, 1832), *Anomala matzenaueri* Reitter, 1918, *Exomala adriatica* (Petrovitz, 1968) and *Oxythyrea dulcis* Reitter, 1899. Thus, the number of currently known scarabaeoid species in Montenegro has increased to 184. Twenty-four species of scarabaeoid beetles are illustrated. Our results indicate insufficient knowledge of the Scarabaeoidea of Montenegro and, at the same time, their diversity and the presence of rare species among them. High-mountain and coastal communities of coprophagic scarabaeoid beetles, as well as communities of scarabaeoid beetles inhabiting coastal dunes, are especially valuable, worthy of protection. Therefore, further research and new expeditions to Montenegro are highly desirable.

## 1. Introduction

The Scarabaeoidea (scarabaeoid beetles) inhabit all zoogeographic regions of the world. They are the most species-rich in the tropics and the further north, the less numerous they become. In Europe, scarabaeoid beetles are represented by 8 families, 208 genera and ca. 1250 species [1]. A dominant trophic group among scarabaeoid beetles are dung beetles. Dung beetles are encountered worldwide, but they are particularly diverse in tropical forests and savannahs [2]. Evidence of fossil faeces clearly indicates that dung beetles had been connected with dinosaurs even before the mammals evolved [3]. Dung beetles can be endocoprid, paracoprid or telecoprid [4]. Endocoprid species lay eggs directly into the dung on the soil surface. Paracropid species dig tunnels beneath or nearby the dung, which end with brood chambers. Telecoprid species separate a portion of dung and transport it far from the original dung source, where the beetles bury in short to deep tunnels [5]. The diversified methods of dung transporting and foraging obviously result in an increased diversification of ecological processes such as nutrient recycling, soil aeration, plant seed dispersal, and reduction of parasite numbers, which provide benefits both to the ecosystem and to human activities [6]. Among the scarabaeoid beetles, there are also saproxylophagous, necrophagous, phyllophagous and rhizophagous species. There are forest and pasture species as well as mountain and lowland species; some of them are attracted by flowers or fermenting fruits, while others to artificial lights.

Taxonomic and faunistic works allow us to understand the behaviour of scarabaeoid beetles fully. Such works provide descriptions of new taxa and classify the organisms, and show their distribution, diversity, and habitat requirements. They constitute the basis for further research, e.g., on the relationships in dung beetles communities and their role in nature.

The Montenegrin fauna of the superfamily Scarabaeoidea is not sufficiently studied. The first comprehensive, relevant work was “Fauna Insectorum Balcanica—Scarabaeoidea”, which included information on the occurrence of 97 species from this superfamily in Montenegro [7]. In the first appendix published by the same author, there are 112 species [8]. In the second appendix, Mikšić [9] lists another six species new to the fauna of Montenegro, but at the same time removes the two species mentioned in the first appendix. Consequently, the number of recorded scarabaeoid beetle species from Montenegro increased to 116. According to the “Katalog der Lamellicornia Jugoslawiens (Insecta-Coleoptera)”, the number of scarabaeoid beetle species known from Montenegro was 122 [10].

The Catalogue of Palaearctic Coleoptera [11] contains one common list of Scarabaeoidea species for Montenegro and Serbia, while the second edition of the same catalogue contains two separate lists of scarabaeoid species for these countries. The second edition contains 163 scarabaeoid species (Geotrupidae—7 species, Trogidae—2, Lucanidae—5, Glaphyridae—1 and Scarabaeidae—148) from Montenegro [1]. There are 94 species of dung beetles among the Scarabaeoidea of Montenegro.

Most of the works on the Scarabaeoidea of Montenegro were published in the last century. Some of these works cover a much larger area than the present territory of Montenegro (e.g., the territory of the former Yugoslavia or the Balkan Peninsula). Consequently, many records have a rather general character without any precise locality data. With this research, we aim to supplement the current information about the distribution of the taxa of the superfamily Scarabaeoidea in the country. We also give advice on the protection of valuable scarabaeoid beetle communities.

## 2. Materials and Methods

**Figure 1:**
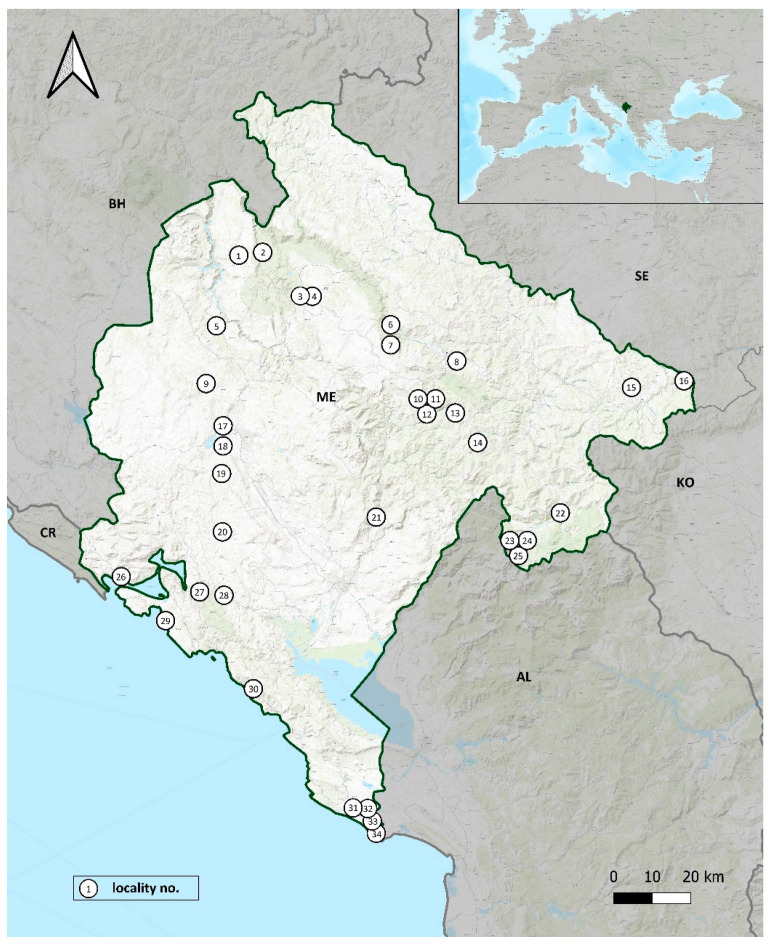
Collection localities of the scarabaeoid beetles in Montenegro (2019, 2021) (numbers of the localities correspond to those in Table 1).

The presented data are the result of three entomological expeditions. The collecting of scarabaeoid beetles was done between 27 June and 2 July 2019, next between 19 May and 2 June, and finally between 17 and 23 July 2021. We collected material in 34 localities (Table 1 and Figure 1) located in fifteen municipalities: Andrijevica (1 locality), Budva (2), Cetynia (2), Gusinje (3), Herceg Novi (1), Kotor (1), Kolašin (4), Mojkovac (3), Nikšić (4), Plav (1), Plužine (2), Podgorica (1), Rožaje (2), Ulcinj (4) and Žabljak (3).

**Table 1:** Collection localities of the scarabaeoid beetles in Montenegro (2019, 2021).

No.	Locality	Geographical Coordinates	Altitude [m a.s.l.]	Date of Collection
1.	Trsa at Plužine (Figure 2A)	43°11′15.2″ N 18°55′54.2″ E	1430	29–30 May 2021
2.	Mala Crna Gora at Žabljak (Figure 2B)	43°11′39.1″ N 19°00′20.9″ E	1545	31 May 2021
3.	Stožina at Žabljak (Figure 2C,D)	43°05′34.4″ N 19°04′30.1″ E	1690	1–2 June 2021
4.	Pošćenski Kraj at Žabljak (Figure 2E)	43°05′39.8″ N 19°06′23.8″ E	1540	31 May–1 June 2021
5.	Bajovo Polje at Plužine	43°01′38.6″ N 18°51′43.9″ E	1100	29 May 2021
6.	Dobrilovina at Mojkovac (Figure 2F)	43°01′35.5″ N 19°24′32.1″ E	715	19 July 2021
7.	Bistrica at Mojkovac	42°59′13.6″ N 19°24′02.3″ E	880	23 July 2021
8.	Brskovo at Mojkovac	42°56′49.8″ N 19°36′38.0″ E	1140	18 July 2021
9.	Donje Srijede at Presjeka (Figure 2G)	42°53′45.3″ N 18°49′48.4″ E	960	28 May 2021
10.	Lipovska Bistrica at Kolašin	42°52′13.1″ N 19°29′15.8″ E	1180	20 July 2021
11.	Radigojno at Kolašin	42°50′44.4″ N 19°32′30.1″ E	1220	18 July 2021
12.	Kolašin	42°49′56.2″ N 19°31′20.1″ E	925	20 July 2021
13.	Muskovica Rijeka at Kolašin	42°49′43.6″ N 19°36′21.3″ E	1285	20 July 2021
14.	Prisojački Katun at Gornje Luge	42°45′40.8″ N 19°40′31.1″ E	1745	22 May 2021
15.	Grahovo at Rožaje (Figure 2H)	42°53′13.6″ N 20°09′16.9″ E	1110	19–20 May 2021
16.	Dračenovac at Rožaje (Figure 3A)	42°54′07.8″ N 20°19′04.3″ E	820	19 May 2021
17.	Crnodoli at Nikšić (Figure 3B)	42°47′42.0″ N 18°52′33.1″ E	630	27–28 May 2021
18.	Riđani at Nikšić (Figure 3C)	42°45′28.9″ N 18°53′22.2″ E	605	28 May 2021
19.	Broćanac Nikšićki at Nikšić (Figure 3D)	42°41′25.0″ N 18°52′41.8″ E	970	27 May 2021
20.	Čevo at Cetynia (Figure 3E)	42°33′23.9″ N 18°52′50.6″ E	780	27 May 2021
21.	Donje Polje at Mrke	42°35′25.5″ N 19°21′33.9″ E	145	22 May 2021
22.	Plav (Figure 3F)	42°36′00.4″ N 19°55′57.9″ E	910	20 May 2021
23.	Škala at Gusinje (Figure 3G)	42°31′51.0″ N 19°47′29.5″ E	1070	21 May 2021
24.	Mayusanje at Gusinje (Figure 3H and Figure 4A)	42°31′48.4″ N 19°50′08.2″ E	930	21–22 May 2021
25.	Grebaje at Gusinje (Figure 4B)	42°30′53.9″ N 19°46′53.5″ E	1170	21 May 2021
26.	Meljine at Herceg Novi	42°27′14.1″ N 18°33′55.7″ E	10	28 June–2 July 2019
27.	Žanjev Do at Njeguši (Figure 4C)	42°25′08.3″ N 18°48′36.2″ E	1120	25 May 2021
28.	Dubovik at Cetynia (Figure 4D,E)	42°24′40.3″ N 18°53′08.2″ E	920	25–27 May 2021
29.	Bigova at Kotor	42°21′10.4″ N 18°42′12.8″ E	20	15–17 July 2021
30.	Buljarica at Petrovac na Moru	42°11′46.1″ N 18°58′36.5″ E	15	25 May 2021
31.	Doni Štoj at Ulcinj (dunes) (Figure 4F)	41°53′12.1″ N 19°18′41.3″ E	0	24 May 2021
32.	Doni Štoj at Ulcinj (pasture)	41°53′19.8″ N 19°20′42.9″ E	0	24 May 2021
33.	Sveti Nikola at Ulcinj	41°52′43.1″ N 19°21′24.0″ E	5	23 May 2021
34.	Bojana at Ulcinj (Figure 4G,H)	41°51′28.1″ N 19°21′09.0″ E	0	23–24 May 2021

**Figure 2:**
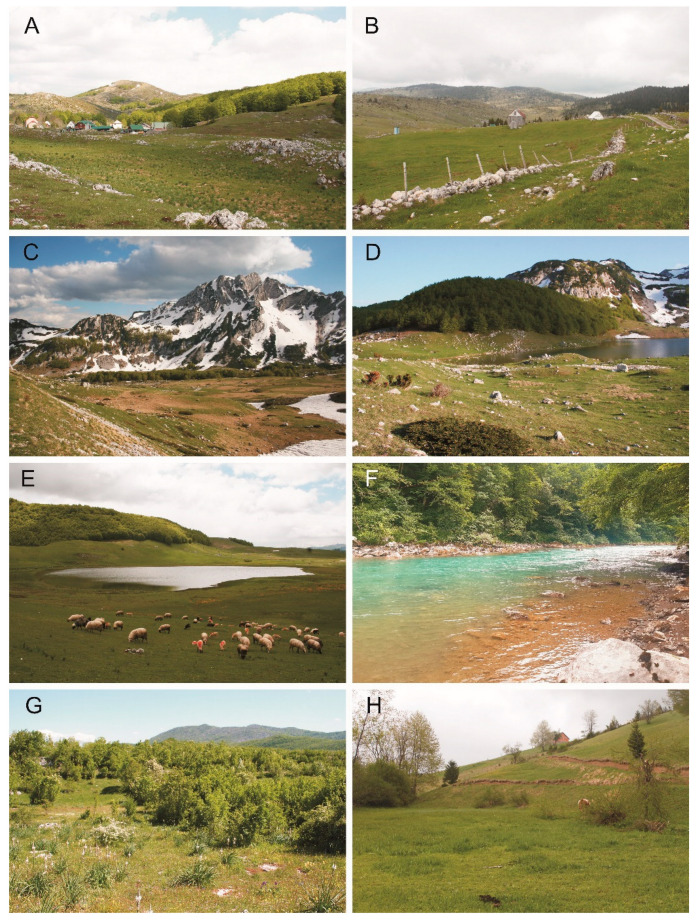
Collection localities of the scarabaeoid beetles in Montenegro (2019, 2021): (**A**)—Trsa at Plužine, (**B**)—Mala Crna Gora at Žabljak, (**C**,**D**)—Stožina at Žabljak, (**E**)—Pošćenski Kraj at Žabljak, (**F**)—Dobrilovina at Mojkovac, (**G**)—Donje Srijede at Presjeka, (**H**)—Grahovo at Rožaje (Photos: (**A**,**C**–**E**,**G**)—Tomasz Gazurek, (**B**,**H**)—Sebastian Tylkowski and (**F**)—Dawid Marczak).

**Figure 3:**
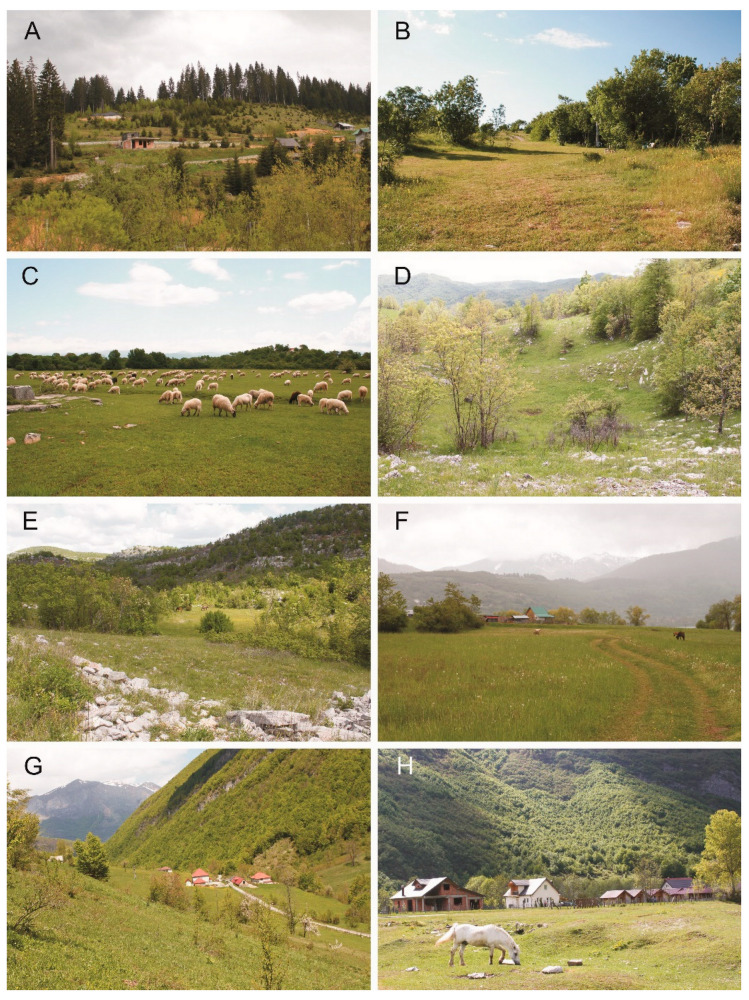
Collection localities of the scarabaeoid beetles in Montenegro (2019, 2021): (**A**)—Dračenovac at Rožaje, (**B**)—Crnodoli at Nikšić, (**C**)—Riđani at Nikšić, (**D**)—Broćanac Nikšićki at Nikšić, (**E**)—Čevo at Cetynia, (**F**)—Plav, (**G**)—Škala at Gusinje, (**H**)—Vusanje at Gusinje (Photos: (**A**,**D**–**H**)—Sebastian Tylkowski and (**B**,**C**)—Tomasz Gazurek).

**Figure 4:**
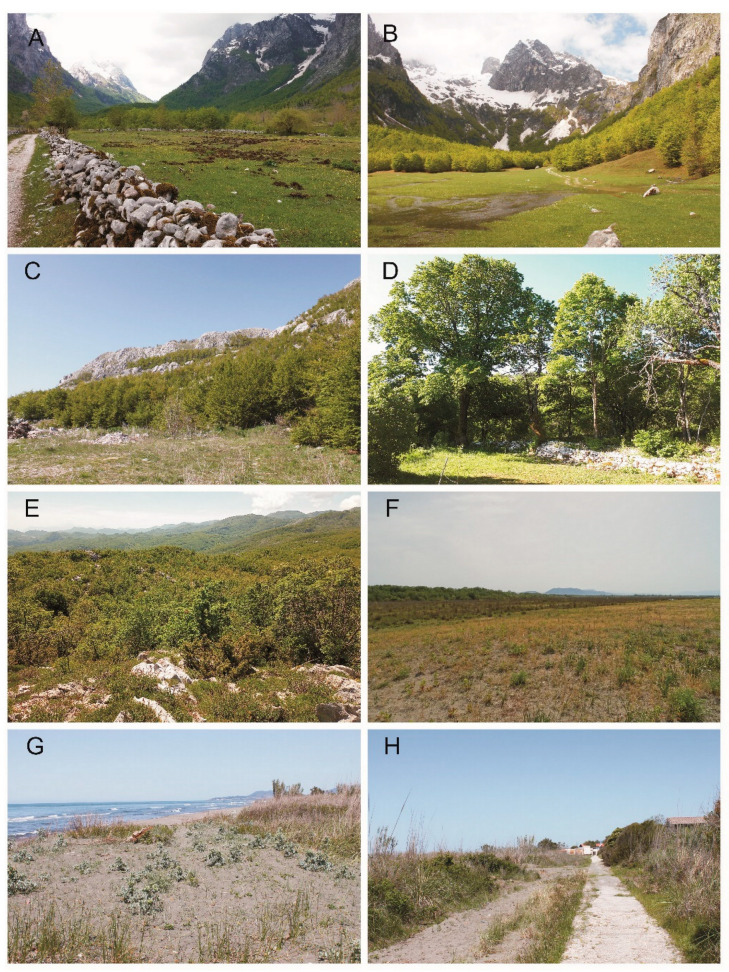
Collection localities of the scarabaeoid beetles in Montenegro (2019, 2021): (**A**)—Vusanje at Gusinje, (**B**)—Grebaje at Gusinje, (**C**)—Žanjev Do at Njeguši, (**D**,**E**)—Dubovik at Cetynia, (**F**)—Doni Štoj at Ulcinj (dunes), (**G**,**H**)—Bojana at Ulcinj (Photos: (**A**)—Tomasz Gazurek, (**B**,**C**,**G**,**H**)—Sebastian Tylkowski, (**D**,**F**)—Adam Byk and (**E**)—Marek Bidas).

During the field studies, a variety of environments were searched and collecting methods were used. We looked for them on flowers, leaves of the trees and grass, on trunks of dead trees and in their hollows, in wood dust, litter, the upper layer of soil, as well as in remnants of dead animals, excrements of domestic and wild animals (e.g., cattles, horses, sheep, dogs, wild boars, foxes). We caught imagines in insect nets, insect sweep nets, insect beating sheets and traps baited with fresh sheep droppings. For psammophilous species, we sifted sand on seashore dunes and riverbanks and examined pieces of wood, stones lying there. Additionally, at night, we attracted insects to UV-illuminated screens and searched on trunks of old trees and their leaves using LED lights.

All the specimens listed below were collected by Marek Bidas [MB], Adam Byk [AB], Tomasz Gazurek [TG], Dawid Marczak [DM] and Sebastian Tylkowski [ST]. Taxa were identified by Marek Bidas [MB], Adam Byk [AB], Tomasz Gazurek [TG] and Łukasz Minkina [ŁM]. Several species were identified or verified by other researchers. Their names are given directly in the list of species below. The specimens are preserved in the entomological collection of the Department of Forest Protection of the Warsaw University of Life Sciences and the private collections of the authors.

Specimens were examined with Nikon SMZ-U and C-PS stereomicroscopes. Photographs of the specimens were taken with Canon EOS 5D Mark III connected with Canon MP-E 65 mm macro lens. Photographs were edited in Helicon Focus and Adobe Photoshop Elements 2018.

The systematic arrangement and nomenclature were adopted from the Catalogue of Palaearctic Coleoptera [1]. Based on the publication of Nikolajev [12], the names of subgenera in the genus *Trox* Fabricius, 1775 were adopted. The authorship of the name of the species *Anoplotrupes stercorosus* has been changed based on the publication of Ziani et al. [13].

## 3. Results

As a result of this study, 2130 beetles belonging to 107 species in five families of the superfamily Scarabaeoidea were collected: Geotrupidae (5 spp.), Trogidae (2 spp.), Lucanidae (2 spp.), Ochodaeidae (1 sp.) and Scarabaeidae (97 spp.): including Aphodiinae (38 spp.), Scarabaeinae (26 spp.), Melolonthinae (11 spp.), Rutelinae (6 spp.), Dynastinae (3 spp.) and Cetoniinae (13 spp.). Among them, 63 species are dung beetles.

The most abundant species of scarabaeoid beetles were: *Esymus pusillus* (107 exx.), *Agrilinus convexus* (100 exx.), *Maladera holosericea* (95 exx.), *Onthophagus verticicornis* (89 exx.), *Melinopterus sphacelatus* (87 exx.), *Onthophagus panici* (77 exx.), *Ammoecius brevis* (73 exx.), *Onthophagus fracticornis* (73 exx.), *Melinopterus prodromus* (68 exx.), *Euoniticellus fulvus* (56 exx.), *Melolontha pectoralis* (54 exx.) and *Aphodius pedellus* (52 exx.). The most commonly observed species of pleurostict scarabaeoid beetles were: *Cetonia aurata* (13 localities) and *Oxythyrea funesta* (12 localities). The laparostict scarabaeoid beetles were dominated by: *Onthophagus fracticornis* with *Aphodius pedellus* (15 localities), *Euoniticellus fulvus* with *Onthophagus taurus* (12 localities), *Esymus pusillus* with *Onthophagus verticicornis* (11 localities), and *O. lemur* with *O. illyricus* (10 localities). Sixteen species are here presented as new records for Montenegro.

The list of the recorded species, along with their new localities, is presented below. Species new to the Montenegro fauna are marked with an asterisk (*).

### List of Taxa

Superfamily SCARABAEOIDEA Latreille, 1802.

Family GEOTRUPIDAE Latreille, 1802.

Subfamily BOLBOCERATINAE Mulsant, 1842.

**Figure 5:**
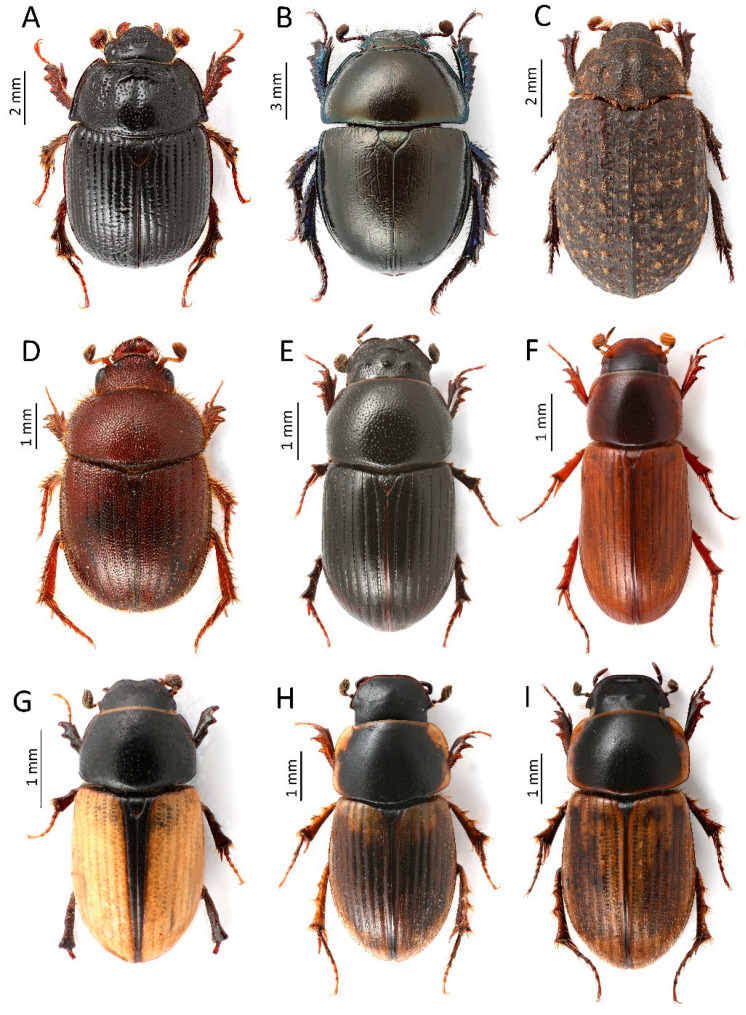
Rare and new species for the fauna of Montenegro were recorded in the course of this study: (**A**)—*Odonteus armiger, (**B**)—*Trypocopris alpinus*, (**C**)—*Trox sabulosus, (**D**)—*Ochodaeus integriceps, (**E**)—*Agrilinus convexus, (**F**)—*Erytus aequalis*, (**G**)—*Euorodalus tersus*, (**H**)—*Melinopterus reyi, (**I**)—*M. sphacelatus (*—species new for the Montenegro fauna) (Photos: Łukasz Minkina).

*Odonteus armiger (Scopoli, 1772) (Figure 5A).

Localities. Crnodoli at Nikšić, 27–28 May 2021, 1 ex. [ST]; Dubovik at Cetynia, 25–27 May 2021, 1 ex. [MB].

Remarks. Reported from most European countries, including those around Montenegro (Albania, Bosnia Herzegovina, Croatia, Serbia), Armenia, Azerbaijan and Turkey [14,15,16,17]. Two individuals of this species were caught with an insect net. One of them was caught at dusk, on a dry and bushy pasture (Figure 3B), the other one at night, on a small clearing in an oak forest (Figure 4D).

Subfamily GEOTRUPINAE Latreille, 1802.

*Anoplotrupes stercorosus* (Hartmann in Scriba, 1791).

Localities. Trsa at Plužine, 29–30 May 2021, 2 exx. [MB], 3 exx. [AB, ST]; Stožina at Žabljak, 1–2 June 2021, 7 exx. [AB, ST]; Pošćenski Kraj at Žabljak, 31 May–1 June 2021, 1 ex. [ST]; Kolašin, 20 July 2021, 1 ex. [DM]; Grahovo at Rožaje, 19–20 May 2021, 6 exx. [AB, ST]; Broćanac Nikšićki at Nikšić, 27 May 2021, 1 ex. [AB]; Vusanje at Gusinje, 21–22 May 2021, 1 ex. [AB, ST].

*Geotrupes mutator* (Marsham, 1802).

Localities. Broćanac Nikšićki at Nikšić, 27 May 2021, 1 ex. [AB, ST]; Dubovik at Cetynia, 25–27 May 2021, 1 ex. [AB].

*Trypocopris alpinus balcanicola* (Mikšić, 1954) (Figure 5B).

Localities. Trsa at Plužine, 29–30 May 2021, 2 exx. [MB], 2 exx. [AB, ST]; Stožina at Žabljak, 1–2 June 2021, 3 exx. [MB], 6 exx. [AB, ST], 5 exx. [TG].

Remarks. Subspecies inhabiting Albania, Bosnia Herzegovina, Italy and Montenegro [16,17,18]. All individuals were caught on high mountain meadows with numerous tunnels dug by rodents (Figure 2A,C). In the morning and at noon, they were caught under the excrement of cows and horses, and in the afternoon, they walked quickly on the meadow.

*Trypocopris vernalis vernalis* (Linnaeus, 1758).

Localities. Trsa at Plužine, 29–30 May 2021, 3 exx. [MB]; Pošćenski Kraj at Žabljak, 31 May–1 June 2021, 1 ex. [MB]; Donje Srijede at Presjeka, 28 May 2021, 1 ex. [AB]; Broćanac Nikšićki at Nikšić, 27 May 2021, 2 exx. [AB, ST]; Žanjev Do at Njeguši, 25 May 2021, 1 ex. [MB]; Dubovik at Cetynia, 25–27 May 2021, 3 exx. [AB, ST].

Family TROGIDAE W.S. Macleay, 1819.

*Trox* (*Granulitrox*) *niger* P. Rossi, 1792.

Localities. Dubovik at Cetynia, 25–27 May 2021, 1 ex. [MB]; Bigova at Kotor, 15–17 July 2021, 2 exx. [DM].

*Trox (Trox) sabulosus sabulosus (Linnaeus, 1758) (Figure 5C).

Localities. Trsa at Plužine, 29–30 May 2021, 3 exx. [MB], 17 exx. [TG]; Mala Crna Gora at Žabljak, 31 May 2021, 10 exx. [AB, ST]; Grebaje at Gusinje, 21 May 2021, 1 ex. [MB], 2 exx. [AB, ST].

Remarks. The subspecies is reported from most European countries (including Montenegro’s neighbours: Albania, Bosnia Herzegovina, Croatia, Serbia), as well as Kazakhstan, Mongolia, North and South Korea, Russia (East and West Siberia), Syria and Turkey [19,20,21]. All individuals were caught on high mountain meadows (Figure 2A,B and Figure 4B). Males and females of this species were caught under the old excrement of dogs and foxes and sheep wool lying on the ground.

Family LUCANIDAE Latreille, 1804.

Subfamily LUCANINAE Latreille, 1804.

Tribe DORCINI Parry, 1864.

*Dorcus parallelipipedus* (Linnaeus, 1758).

Localities. Bistrica at Mojkovac, 23 July 2021, 1 ex. [DM]; Muskovica Rijeka at Kolašin, 20 July 2021, 1 ex. [DM]; Meljine at Herceg Novi, 28 June–2 July 2019, 1 ex. [AB]; Bigova at Kotor, 15–17 July 2021, 1 ex. [DM]; Bojana at Ulcinj, 23–24 May 2021, 2 exx. [AB, ST].

Tribe LUCANINI Latreille, 1804.

*Lucanus cervus cervus* (Linnaeus, 1758).

Localities. Dobrilovina at Mojkovac, 19 July 2021, 1 ex. [DM]; Crnodoli at Nikšić, 27–28 May 2021, 1 ex. (dead) [TG]; Bigova at Kotor, 15–17 July 2021, 1 ex. [DM].

Family OCHODAEIDAE Mulsant & Rey, 1871.

*Ochodaeus integriceps Semenov, 1891 (Figure 5D).

Locality. Bojana at Ulcinj, 23–24 May 2021, 1 ex. [MB], 3 exx. [AB, ST], 1 ex. [TG].

Remarks. Known from many countries of Europe (Armenia, Austria, Azerbaijan, Bulgaria, Czech Republic, Croatia, Georgia, Hungary, North Macedonia, south of European part of Russia, Serbia, Slovakia, Ukraine) [22]. All individuals of this species were caught with an insect net. They flew at dusk and in the early night among tall reeds adjacent to the coastal dunes (Figure 4G).

Family SCARABAEIDAE Latreille, 1802.

Subfamily APHODIINAE Leach, 1815.

Tribe APHODIINI Leach, 1815.

Subtribe APHODIINA Leach, 1815.

*Acanthobodilus immundus* (Creutzer, 1799).

Localities. Bigova at Kotor, 15–17 July 2021, 1 ex. [DM]; Doni Štoj at Ulcinj (dunes), 24 May 2021, 2 exx. [AB, ST]; Sveti Nikola at Ulcinj, 23 May 2021, 2 exx. [MB], 2 exx. [AB, ST].

*Acrossus depressus* (Kugelann, 1792).

Localities. Stožina at Žabljak, 1–2 June 2021, 5 exx. [AB, ST]; Pošćenski Kraj at Žabljak, 31 May–1 June 2021, 2 exx. [MB], 6 exx. [AB, ST]; Grahovo at Rožaje, 19–20 May 2021, 2 exx. [MB], 7 exx. [AB, ST]; Broćanac Nikšićki at Nikšić, 27 May 2021, 1 ex. [ST]; Škala at Gusinje, 21 May 2021, 1 ex. [MB], 3 exx. [AB]; Dubovik at Cetynia, 25–27 May 2021, 4 exx. [AB, ST].

*Acrossus luridus* (Fabricius, 1775).

Localities. Trsa at Plužine, 29–30 May 2021, 2 exx. [MB], 3 exx. [AB, ST]; Stožina at Žabljak, 1–2 June 2021, 5 exx. [AB, ST]; Pošćenski Kraj at Žabljak, 31 May–1 June 2021, 3 exx. [AB, ST]; Riđani at Nikšić, 28 May 2021, 3 exx. [MB], 6 exx. [AB, ST]; Broćanac Nikšićki at Nikšić, 27 May 2021, 1 ex. [ST]; Čevo at Cetynia, 27 May 2021, 2 exx. [AB, ST]; Plav, 20 May 2021, 3 exx. [MB], 12 exx. [AB, ST]; Škala at Gusinje, 21 May 2021, 4 exx. [AB, ST]; Vusanje at Gusinje, 21–22 May 2021, 1 ex. [AB].

*Agrilinus convexus (Erichson, 1848) (Figure 5E).

Localities. Trsa at Plužine, 29–30 May 2021, 6 exx. [MB], 22 exx. [AB, ST]; Stožina at Žabljak, 1–2 June 2021, 2 exx. [AB, ST]; Pošćenski Kraj at Žabljak, 31 May–1 June 2021, 12 exx. [MB], 24 exx. [AB, ST]; Grahovo at Rožaje, 19–20 May 2021, 3 exx. [MB], 23 exx. [AB, ST]; Plav, 20 May 2021, 1 ex. [MB], 7 exx. [AB, ST].

Remarks. Known from many European countries (including Montenegro’s neighbours: Albania, Bosnia Herzegovina, Croatia, Serbia), as well as Armenia, Azerbaijan and Georgia. Recorded also in Iran, Mongolia, Asiatic Russia (East and West Siberia, Far East), Turkey, Algeria, Morocco and Tunisia [17,23,24,25]. Imagines were caught in sheep, cow and horse dung in mountain pastures (over 900 m above sea level) (Figure 2A,D,E,H and Figure 3F).

*Amidorus obscurus obscurus* (Fabricius, 1792).

Locality. Stožina at Žabljak, 1–2 June 2021, 1 ex. [AB].

*Ammoecius brevis* (Erichson, 1848).

Localities. Trsa at Plužine, 29–30 May 2021, 28 exx. [MB], 44 exx. [AB, ST]; Mala Crna Gora at Žabljak, 31 May 2021, 1 ex. [AB].

*Aphodius coniugatus* (Panzer, 1795).

Locality. Trsa at Plužine, 29–30 May 2021, 2 exx. [AB, ST].

*Aphodius fimetarius* (Linnaeus, 1758).

Localities. Doni Štoj at Ulcinj (dunes), 24 May 2021, 6 exx. [AB, ST]; Doni Štoj at Ulcinj (pasture), 24 May 2021, 10 exx. [MB]; Bojana at Ulcinj, 23–24 May 2021, 2 exx. [AB, ST], 2 exx. [TG].

*Aphodius foetidus* (Herbst, 1783).

Locality. Buljarica at Petrovac na Moru, 25 May 2021, 1 ex. [AB].

*Aphodius pedellus* (De Geer, 1774).

Localities. Trsa at Plužine, 29–30 May 2021, 2 exx. [MB], 7 exx. [AB, ST]; Mala Crna Gora at Žabljak, 31 May 2021, 1 ex. [AB]; Stožina at Žabljak, 1–2 June 2021, 2 exx. [MB], 1 ex. [TG]; Pošćenski Kraj at Žabljak, 31 May–1 June 2021, 6 exx. [AB, ST]; Bajovo Polje at Plužine, 29 May 2021, 2 exx. [AB, ST]; Donje Srijede at Presjeka, 28 May 2021, 1 ex. [ST]; Kolašin, 20 July 2021, 7 exx. [DM]; Prisojački Katun at Gornje Luge, 22 May 2021, 1 ex. [MB]; Grahovo at Rožaje, 19–20 May 2021, 4 exx. [AB, ST]; Crnodoli at Nikšić, 27–28 May 2021, 1 ex. [MB], 1 ex. [ST]; Broćanac Nikšićki at Nikšić, 27 May 2021, 4 exx. [AB, ST]; Plav, 20 May 2021, 1 ex. [MB], 7 exx. [AB, ST]; Škala at Gusinje, 21 May 2021, 1 ex. [MB]; Žanjev Do at Njeguši, 25 May 2021, 2 exx. [AB, ST]; Bigova at Kotor, 15–17 July 2021, 1 ex. [DM].

*Calamosternus granarius* (Linnaeus, 1767).

Localities. Pošćenski Kraj at Žabljak, 31 May–1 June 2021, 2 exx. [MB]; Kolašin, 20 July 2021, 4 exx. [DM]; Crnodoli at Nikšić, 27–28 May 2021, 3 exx. [AB, ST], 1 ex. [TG]; Škala at Gusinje, 21 May 2021, 1 ex. [AB]; Žanjev Do at Njeguši, 25 May 2021, 1 ex. [AB]; Dubovik at Cetynia, 25–27 May 2021, 2 exx. [AB, ST]; Bojana at Ulcinj, 41°51′28.1″ N 19°21′09.0″ E, 0 m a.s.l., 23–24 May 2021, 2 exx. [TG].

*Colobopterus erraticus* (Linnaeus, 1758).

Localities. Radigojno at Kolašin, 18 July 2021, 1 ex. [DM]; Kolašin, 20 July 2021, 1 ex. [DM]; Grahovo at Rožaje, 19–20 May 2021, 9 exx. [AB, ST]; Riđani at Nikšić, 28 May 2021, 4 exx. [AB, ST]; Broćanac Nikšićki at Nikšić, 27 May 2021, 2 exx. [AB, ST]; Plav, 20 May 2021, 8 exx. [AB, ST]; Vusanje at Gusinje, 21–22 May 2021, 3 exx. [AB, ST]; Doni Štoj at Ulcinj (dunes), 24 May 2021, 2 exx. [AB, ST].

*Coprimorphus scrutator* (Herbst, 1789).

Locality. Kolašin, 20 July 2021, 2 exx. [DM].

*Erytus aequalis* (A. Schmidt, 1907) (Figure 5F).

Locality. Doni Štoj at Ulcinj (pasture), 24 May 2021, 7 exx. [MB], 20 exx. [AB, ST].

Remarks. Central Asian-European species. Distribution in Asia extends east to northwestern China (Xinjiang and Xizang). In Europe, the species is reported from Albania, Bulgaria, France (Corsica), Italy, Montenegro, Romania and Greece, central and south of the European parts of Russia, Ukraine, as well as Armenia, Azerbaijan and Georgia. In Africa, it is reported from Algeria, Libya and Tunisia [25,26]. Found in Albania and Montenegro for the first time in 2015 [26]. All individuals of this species were caught in sheep droppings on a highly sunlit pasture, with a large number of bee-eater burrows. This is the second record from Montenegro.

*Esymus merdarius* (Fabricius, 1775).

Localities. Trsa at Plužine, 29–30 May 2021, 2 exx. [MB]; Vusanje at Gusinje, 21–22 May 2021, 1 ex. [MB].

*Esymus pusillus pusillus* (Herbst, 1789).

Localities. Trsa at Plužine, 29–30 May 2021, 13 exx. [MB], 19 exx. [AB, ST], 5 exx. [TG]; Mala Crna Gora at Žabljak, 31 May 2021, 3 exx. [AB, ST]; Stožina at Žabljak, 1–2 June 2021, 6 exx. [AB, ST]; Pošćenski Kraj at Žabljak, 31 May–1 June 2021, 15 exx. [AB, ST]; Bajovo Polje at Plužine, 29 May 2021, 1 ex. [MB]; Grahovo at Rožaje, 19–20 May 2021, 8 exx. [AB, ST]; Broćanac Nikšićki at Nikšić, 27 May 2021, 4 exx. [AB, ST]; Plav, 20 May 2021, 1 ex. [MB], 20 exx. [AB, ST]; Škala at Gusinje, 21 May 2021, 1 ex. [MB], 4 exx. [AB, ST]; Vusanje at Gusinje, 21–22 May 2021, 1 ex. [MB], 3 exx. [AB, ST]; Doni Štoj at Ulcinj (dunes), 24 May 2021, 3 exx. [AB, ST].

*Eudolus quadriguttatus* (Herbst, 1783).

Localities. Crnodoli at Nikšić, 27–28 May 2021, 1 ex. [MB]; Riđani at Nikšić, 28 May 2021, 1 ex. [AB]; Doni Štoj at Ulcinj (pasture), 24 May 2021, 2 exx. [MB], 5 exx. [AB, ST].

*Euorodalus paracoenosus* (Balthasar & Hrubant, 1960).

Localities. Donje Srijede at Presjeka, 28 May 2021, 1 ex. [ST]; Čevo at Cetynia, 27 May 2021, 1 ex. [AB]; Plav, 20 May 2021, 1 ex. [AB]; Škala at Gusinje, 21 May 2021, 1 ex. [ST].

*Euorodalus tersus* (Erichson, 1848) (Figure 5G).

Locality. Sveti Nikola at Ulcinj, 23 May 2021, 1 ex. [MB].

Remarks. Mediterranean species are known from five countries in Africa (Algeria, Egypt, Libya, Morocco, Tunisia) and six countries in Europe (Albania, Italy, Malta, Montenegro, Portugal, Spain) [25,26]. Found in Albania and Montenegro for the first time in 2015 [26]. One individual of this species was caught in the dung of a cow on a pasture. This is the second record from Montenegro.

*Melinopterus consputus* (Creutzer, 1799).

Localities. Vusanje at Gusinje, 21–22 May 2021, 1 ex. [MB]; Bigova at Kotor, 15–17 July 2021, 4 exx. [DM].

*Melinopterus prodromus* (Brahm, 1790).

Localities. Stožina at Žabljak, 1–2 June 2021, 3 exx. [AB, ST]; Pošćenski Kraj at Žabljak, 31 May–1 June 2021, 1 ex. [MB], 14 exx. [AB, ST]; Grahovo at Rožaje, 19–20 May 2021, 1 ex. [AB]; Dračenovac at Rožaje, 19 May 2021, 4 exx. [MB]; Plav, 20 May 2021, 9 exx. [MB], 11 exx. [AB, ST]; Škala at Gusinje, 21 May 2021, 7 exx. [AB, ST]; Vusanje at Gusinje, 21–22 May 2021, 3 exx. [MB], 12 exx. [AB, ST]; Dubovik at Cetynia, 25–27 May 2021, 1 ex. [MB], 2 exx. [AB, ST].

*Melinopterus reyi (Reitter, 1892) (Figure 5H).

Locality. Dračenovac at Rožaje, 19 May 2021, 4 exx. [MB];

Remarks. A species widely distributed in Europe. Reported from Austria, Bosnia Herzegovina, Bulgaria, Czech Republic, Denmark, France, Germany, Hungary, Italy, Luxembourg, The Netherlands, Poland, Romania, the southern part of the European territory of Russia, Slovakia, Spain, Switzerland, European and Asian parts of Turkey and Ukraine. Among the countries bordering Montenegro, found only in Bosnia Herzegovina [25,27]. All individuals of this species were caught in cow dung lying on the road near the river (Figure 3A).

*Melinopterus sphacelatus (Panzer, 1798) (Figure 5I).

Localities. Trsa at Plužine, 29–30 May 2021, 6 exx. [MB], 14 exx. [AB, ST]; Stožina at Žabljak, 1–2 June 2021, 10 exx. [AB, ST]; Pošćenski Kraj at Žabljak, 31 May–1 June 2021, 10 exx. [MB], 19 exx. [AB, ST]; Grahovo at Rožaje, 19–20 May 2021, 1 ex. [MB]; Škala at Gusinje, 21 May 2021, 8 exx. [AB, ST]; Vusanje at Gusinje, 21–22 May 2021, 3 exx. [MB], 16 exx. [AB, ST].

Remarks. Known from many European countries (including Montenegro’s neighbours: Albania, Bosnia Herzegovina, Croatia, Serbia), as well as Armenia, Azerbaijan and Georgia. Reported in Asia from Iraq, Israel, Kazakhstan, Kyrgyzstan, Russia (East and West Siberia), Turkey, Turkmenistan, and from North Africa in Algeria, Morocco and Tunisia [25]. Imagines were caught in cow and horse dung on mountain pastures (over 900 m above sea level) (Figure 2A,D,E,H and Figure 3G,H).

*Nialus varians* (Duftschmidt, 1805).

Localities. Radigojno at Kolašin, 18 July 2021, 3 exx. [DM]; Dračenovac at Rožaje, 19 May 2021, 1 ex. [MB]; Crnodoli at Nikšić, 27–28 May 2021, 2 exx. [AB, ST]; Riđani at Nikšić, 28 May 2021, 2 exx. [MB], 5 exx. [AB, ST]; Plav, 20 May 2021, 3 exx. [MB], 6 exx. [AB, ST]; Škala at Gusinje, 21 May 2021, 6 exx. [AB, ST]; Vusanje at Gusinje, 21–22 May 2021, 1 ex. [ST]; Bojana at Ulcinj, 23–24 May 2021, 6 exx. [AB, ST], 1 ex. [TG].

*Otophorus haemorrhoidalis* (Linnaeus, 1758).

Localities. Trsa at Plužine, 29–30 May 2021, 3 exx. [MB], 5 exx. [AB, ST]; Grahovo at Rožaje, 19–20 May 2021, 1 ex. [AB]; Doni Štoj at Ulcinj (dunes), 24 May 2021, 2 exx. [AB, ST]; Sveti Nikola at Ulcinj, 23 May 2021, 1 ex. [AB]; Bojana at Ulcinj, 23–24 May 2021, 2 exx. [AB, ST].

*Oxyomus sylvestris* (Scopoli, 1763).

Localities. Crnodoli at Nikšić, 27–28 May 2021, 1 ex. [TG]; Plav, 20 May 2021, 1 ex. [AB].

**Figure 6:**
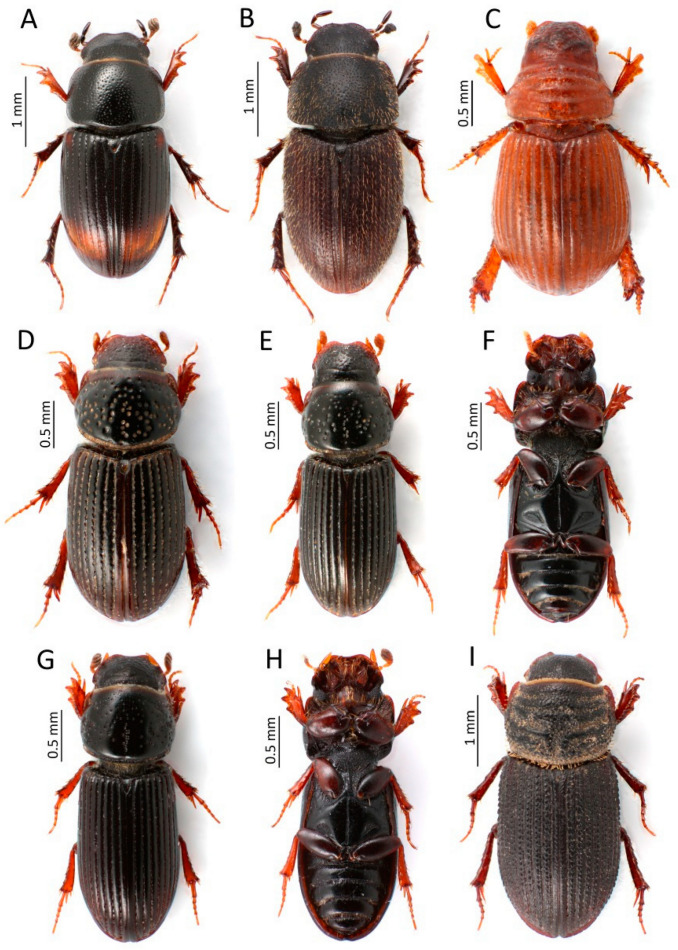
Rare and new species for the fauna of Montenegro recorded in the course of this study: (**A**)—*Phalacronothus biguttatus, (**B**)—*Trichonotulus scrofa, (**C**)—*Psammodius nocturnus, (**D**)—*Platytomus tibialis, (**E**,**F**)—*Pleurophorus mediterranicus, (**G**,**H**)—*P. pannonicus, (**I**)—*Rhyssemus berytensis (*—species new for the Montenegro fauna) (Photos: Łukasz Minkina).

*Phalacronothus biguttatus (Germar, 1824) (Figure 6A).

Localities. Riđani at Nikšić, 28 May 2021, 1 ex. [MB]; Škala at Gusinje, 21 May 2021, 4 exx. [MB], 6 exx. [AB, ST]; Vusanje at Gusinje, 21–22 May 2021, 1 ex. [ST]; Grebaje at Gusinje, 21 May 2021, 7 exx. [MB].

Remarks. Known from many European countries (including Montenegro’s neighbours: Albania, Bosnia Herzegovina, Croatia, Serbia), as well as Armenia and Georgia. Reported from Kazakhstan, Asiatic Russia (West Siberia), Turkey, Algeria, Morocco and Tunisia [25]. Collected from underneath sheep droppings on pastures (Figure 3C,G and Figure 4A,B).

*Phalacronothus quadrimaculatus* (Linnaeus, 1760).

Localities. Trsa at Plužine, 29–30 May 2021, 1 ex. [MB], 3 exx. [AB, ST]; Pošćenski Kraj at Žabljak, 31 May–1 June 2021, 5 exx. [MB], 2 exx. [AB, ST]; Kolašin, 20 July 2021, 2 exx. [DM].

*Plagiogonus arenarius* (A.G. Olivier, 1789).

Locality. Žanjev Do at Njeguši, 25 May 2021, 1 ex. [AB].

*Subrinus sturmi* (Harold, 1870).

Locality. Bojana at Ulcinj, 23–24 May 2021, 1 ex. [AB].

*Teuchestes fossor* (Linnaeus, 1758).

Localities. Trsa at Plužine, 29–30 May 2021, 1 ex. [MB], 5 exx. [AB, ST]; Mala Crna Gora at Žabljak, 31 May 2021, 1 ex. [ST]; Pošćenski Kraj at Žabljak, 31 May–1 June 2021, 1 ex. [AB]; Bajovo Polje at Plužine, 29 May 2021, 1 ex. [AB]; Radigojno at Kolašin, 18 July 2021, 1 ex. [DM]; Kolašin, 20 July 2021, 1 ex. [DM]; Grahovo at Rožaje, 19–20 May 2021, 1 ex. [MB], 2 exx. [AB, ST]; Škala at Gusinje, 21 May 2021, 6 exx. [AB, ST]; Žanjev Do at Njeguši, 25 May 2021, 1 ex. [ST].

*Trichonotulus scrofa (Fabricius, 1787) (Figure 6B).

Localities. Donje Srijede at Presjeka, 28 May 2021, 1 ex. [MB]; Riđani at Nikšić, 28 May 2021, 2 exx. [MB], 3 exx. [AB, ST]; Plav, 20 May 2021, 6 exx. [AB, ST]; Škala at Gusinje, 21 May 2021, 2 exx. [AB, ST]; Vusanje at Gusinje, 21–22 May 2021, 1 ex. [MB], 11 exx. [AB, ST].

Remarks. A species widely distributed in Europe (from Portugal to the European part of Russia, including Montenegro’s neighbours: Albania, Bosnia Herzegovina, Croatia, Serbia), Caucasus, Central and Eastern Asia. Recorded also in Morocco. Accidentally introduced into Canada and the USA [25,28,29,30]. Collected from underneath sheep droppings on pastures (Figure 2G and Figure 3C,F–H).

*Volinus sticticus* (Panzer, 1798).

Localities. Grahovo at Rožaje, 19–20 May 2021, 7 exx. [AB, ST]; Dračenovac at Rožaje, 19 May 2021, 1 ex. [MB]; Crnodoli at Nikšić, 27–28 May 2021, 2 exx. [AB, ST]; Broćanac Nikšićki at Nikšić, 27 May 2021, 3 exx. [AB, ST]; Škala at Gusinje, 21 May 2021, 6 exx. [AB, ST]; Vusanje at Gusinje, 21–22 May 2021, 5 exx. [AB, ST]; Dubovik at Cetynia, 25–27 May 2021, 4 exx. [AB, ST].

Tribe PSAMMODIINI Mulsant, 1842.

Subtribe PSAMMODIINA Mulsant, 1842.

*Psammodius nocturnus Reitter, 1892 (Figure 6C).

Locality. Doni Štoj at Ulcinj (dunes), 24 May 2021, 4 exx. [MB], 1 ex. [AB].

Remarks. Mediterranean species known from four countries in Asia (Cyprus, Israel, Lebanon, Turkey), two countries in Africa (Algeria, Tunisia) and two countries in Europe (Greece, Italy) [31,32]. All individuals of this species were found between the roots of grasses growing on seashore dunes, at a depth of about 30 cm between dry and moist sand (Figure 4F).

Subtribe RHYSSEMINA Pittino & Mariani, 1986.

*Platytomus tibialis (Fabricius, 1798) (Figure 6D).

Locality. Dobrilovina at Mojkovac, 19 July 2021, 1 ex. [DM].

Remarks. Species inhabiting Europe (Albania, Bosnia Herzegovina, Croatia, France, Greece, Italy, Malta and Romania), Asia (Cyprus, Israel, Lebanon, Syria, Turkey) and Africa (Algeria, Canary Islands, Egypt, Libya, Morocco, Madeira Archipelago, Tunisia). Accidentally introduced into Australia, the Azores and the USA [31,32]. Found under a stone on the bank of the Tara River (Figure 2F).

*Pleurophorus mediterranicus Pittino & Mariani, 1986 (Figure 6E,F).

Locality. Dobrilovina at Mojkovac, 19 July 2021, 2 exx. [DM].

Remarks. The species is known in eight European (Belgium, Bosnia Herzegovina, France, Germany, Greece, Italy, Portugal and Spain) and three North African countries (Algeria, Morocco, Tunisia) [32]. Found under the stones on the bank of the Tara River (Figure 2F).

*Pleurophorus pannonicus Petrovitz, 1961 (Figure 6G,H).

Locality. Bojana at Ulcinj, 23–24 May 2021, 5 exx. [TG].

Remarks. A species widely distributed in Europe. Reported from Austria, Belgium, Bosnia Herzegovina, Bulgaria, Croatia, Czech Republic, France, Greece, Hungary, Italy, North Macedonia, Romania, the southern part of the European territory of Russia, Slovakia, Slovenia, Switzerland, Ukraine, as well as Azerbaijan. Among the countries bordering Montenegro, it is found only in Bosnia and Herzegovina. In Asia, reported from Cyprus, Iran, Turkey and Uzbekistan [32]. All the specimens were caught in the afternoon on the sidewalk in a holiday resort located by the sea (Figure 4H). They were caught in an insect sweep net.

*Rhyssemus berytensis Marseul, 1878 (Figure 6I).

Localities. Doni Štoj at Ulcinj (dunes), 24 May 2021, 1 ex. [AB]; Bojana at Ulcinj, 23–24 May 2021, 5 exx. [MB], 6 exx. [AB, ST], 16 exx. [TG].

Remarks. In Europe, it is only known in Bulgaria and Greece (Macedonia) [32,33]. Outside Europe, it is reported in Cyprus, Iran, Iraq, Israel, Lebanon and Turkey. Individuals of this species were observed in the evening between 5 p.m. and 8 p.m. Most of them were found on the sidewalk in a holiday resort located by the sea, and single ones between the roots of grasses growing on seashore dunes (Figure 4F,H).

Subfamily SCARABAEINAE Latreille, 1802.

Tribe COPRINI Leach, 1815.

*Copris hispanus cavolinii* (V. Petagna, 1792).

Locality. Bigova at Kotor, 15–17 July 2021, 2 exx. [DM].

*Copris lunaris* (Linnaeus, 1758).

Localities. Trsa at Plužine, 29–30 May 2021, 2 exx. [AB, ST]; Bajovo Polje at Plužine, 29 May 2021, 2 exx. [AB, ST]; Donje Srijede at Presjeka, 28 May 2021, 2 exx. [AB, ST]; Radigojno at Kolašin, 18 July 2021, 1 ex. [DM]; Kolašin, 20 July 2021, 1 ex. [DM]; Broćanac Nikšićki at Nikšić, 27 May 2021, 4 exx. [AB, ST]; Škala at Gusinje, 21 May 2021, 2 exx. [AB, ST]; Dubovik at Cetynia, 25–27 May 2021, 3 exx. [AB, ST].

Tribe GYMNOPLEURINI Lacordaire, 1856.

*Gymnopleurus sturmii* (W.S. Mcleay, 1821).

Localities. Radigojno at Kolašin, 18 July 2021, 1 ex. [DM]; Bigova at Kotor, 15–17 July 2021, 3 exx. [DM].

Tribe ONITICELLINI H.J. Kolbe, 1905.

*Euoniticellus fulvus* (Goeze, 1777).

Localities. Donje Srijede at Presjeka, 28 May 2021, 3 exx. [AB, ST]; Radigojno at Kolašin, 18 July 2021, 21 exx. [DM]; Kolašin, 20 July 2021, 3 exx. [DM]; Crnodoli at Nikšić, 27–28 May 2021, 3 exx. [AB, ST]; Riđani at Nikšić, 28 May 2021, 1 ex. [AB]; Broćanac Nikšićki at Nikšić, 27 May 2021, 4 exx. [AB, ST]; Čevo at Cetynia, 27 May 2021, 5 exx. [AB, ST]; Plav, 20 May 2021, 2 exx. [MB], 4 exx. [AB, ST]; Žanjev Do at Njeguši, 25 May 2021, 3 exx. [AB, ST]; Buljarica at Petrovac na Moru, 25 May 2021, 3 exx. [AB, ST]; Doni Štoj at Ulcinj (pasture), 24 May 2021, 2 exx. [AB, ST]; Bojana at Ulcinj, 23–24 May 2021, 2 exx. [AB, ST].

*Euoniticellus pallipes* (Fabricius, 1781).

Localities. Kolašin, 20 July 2021, 2 exx. [DM]; Doni Štoj at Ulcinj (dunes), 24 May 2021, 1 ex. [MB]; Doni Štoj at Ulcinj (pasture), 24 May 2021, 1 ex. [AB]; Bojana at Ulcinj, 23–24 May 2021, 7 exx. [MB], 3 exx. [AB, ST], 3 exx. [TG].

Tribe ONTHOPHAGINI Burmeister, 1846.

*Caccobius schreberi* (Linnaeus, 1767).

Localities. Donje Srijede at Presjeka, 28 May 2021, 2 exx. [AB, ST]; Radigojno at Kolašin, 18 July 2021, 3 exx. [DM]; Riđani at Nikšić, 28 May 2021, 1 ex. [MB]; Čevo at Cetynia, 27 May 2021, 5 exx. [AB, ST]; Plav, 20 May 2021, 1 ex. [MB], 3 exx. [AB, ST]; Žanjev Do at Njeguši, 25 May 2021, 1 ex. [ST]; Bigova at Kotor, 15–17 July 2021, 1 ex. [DM]; Buljarica at Petrovac na Moru, 25 May 2021, 2 exx. [AB, ST].

*Euonthophagus amyntas alces* (Fabricius, 1792).

Localities. Buljarica at Petrovac na Moru, 25 May 2021, 2 exx. [MB], 2 exx. [AB, ST]; Doni Štoj at Ulcinj (dunes), 24 May 2021, 2 exx. [AB, ST].

*Onthophagus (Furconthophagus) furcatus* (Fabricius, 1781).

Localities. Donje Polje at Mrke, 22 May 2021, 1 ex. [ST]; Buljarica at Petrovac na Moru, 25 May 2021, 2 exx. [AB, ST]; Doni Štoj at Ulcinj (dunes), 24 May 2021, 4 exx. [AB, ST]; Doni Štoj at Ulcinj (pasture), 24 May 2021, 2 exx. [AB, ST]; Sveti Nikola at Ulcinj, 23 May 2021, 2 exx. [MB], 3 exx. [AB, ST]; Bojana at Ulcinj, 23–24 May 2021, 2 exx. [AB, ST], 4 exx. [TG].

*Onthophagus (Onthophagus) illyricus* (Scopoli, 1763).

Localities. Bajovo Polje at Plužine, 29 May 2021, 2 exx. [AB, ST]; Donje Srijede at Presjeka, 28 May 2021, 4 exx. [AB, ST]; Radigojno at Kolašin, 18 July 2021, 2 exx. [DM]; Kolašin, 20 July 2021, 1 ex. [DM]; Crnodoli at Nikšić, 27–28 May 2021, 1 ex. [AB]; Broćanac Nikšićki at Nikšić, 27 May 2021, 3 exx. [AB, ST]; Čevo at Cetynia, 27 May 2021, 3 exx. [AB, ST]; Plav, 20 May 2021, 3 exx. [AB, ST]; Žanjev Do at Njeguši, 25 May 2021, 3 exx. [AB, ST]; Dubovik at Cetynia, 25–27 May 2021, 2 exx. [AB, ST].

*Onthophagus (Onthophagus) taurus* (Schreber, 1759).

Localities. Trsa at Plužine, 29–30 May 2021, 1 ex. [MB]; Radigojno at Kolašin, 18 July 2021, 2 exx. [DM]; Kolašin, 20 July 2021, 2 exx. [DM]; Crnodoli at Nikšić, 27–28 May 2021, 1 ex. [ST]; Riđani at Nikšić, 28 May 2021, 1 ex. [AB]; Donje Polje at Mrke, 22 May 2021, 4 exx. [AB, ST]; Škala at Gusinje, 21 May 2021, 1 ex. [MB]; Bigova at Kotor, 15–17 July 2021, 6 exx. [DM]; Buljarica at Petrovac na Moru, 25 May 2021, 2 exx. [AB, ST]; Doni Štoj at Ulcinj (dunes), 24 May 2021, 3 exx. [AB, ST]; Doni Štoj at Ulcinj (pasture), 24 May 2021, 2 exx. [AB, ST]; Bojana at Ulcinj, 23–24 May 2021, 3 exx. [AB, ST].

*Onthophagus (Palaeonthophagus) coenobita* (Herbst, 1783).

Localities. Grahovo at Rožaje, 19–20 May 2021, 1 ex. [AB]; Broćanac Nikšićki at Nikšić, 27 May 2021, 1 ex. [AB]; Vusanje at Gusinje, 21–22 May 2021, 1 ex. [MB]; Žanjev Do at Njeguši, 25 May 2021, 1 ex. [ST]; Dubovik at Cetynia, 25–27 May 2021, 3 exx. [AB, ST].

*Onthophagus (Palaeonthophagus) fissicornis* (Steven, 1809).

Locality. Buljarica at Petrovac na Moru, 25 May 2021, 1 ex. [MB], 7 exx. [AB, ST].

*Onthophagus (Palaeonthophagus) fracticornis* (Preyssler, 1790).

Localities. Trsa at Plužine, 29–30 May 2021, 1 ex. [MB], 7 exx. [AB, ST]; Mala Crna Gora at Žabljak, 31 May 2021, 2 exx. [AB, ST]; Stožina at Žabljak, 1–2 June 2021, 17 exx. [AB, ST], 1 ex. [TG]; Pošćenski Kraj at Žabljak, 31 May–1 June 2021, 2 exx. [MB], 7 exx. [AB, ST]; Bajovo Polje at Plužine, 29 May 2021, 1 ex. [ST]; Donje Srijede at Presjeka, 28 May 2021, 8 exx. [AB, ST]; Grahovo at Rožaje, 19–20 May 2021, 7 exx. [AB, ST]; Broćanac Nikšićki at Nikšić, 27 May 2021, 3 exx. [AB, ST]; Čevo at Cetynia, 27 May 2021, 2 exx. [AB, ST]; Plav, 20 May 2021, 1 ex. [MB], 5 exx. [AB, ST]; Škala at Gusinje, 21 May 2021, 2 exx. [AB, ST]; Vusanje at Gusinje, 21–22 May 2021, 4 exx. [AB, ST]; Grebaje at Gusinje, 21 May 2021, 1 ex. [ST]; Žanjev Do at Njeguši, 25 May 2021, 1 ex. [AB]; Dubovik at Cetynia, 25–27 May 2021, 1 ex. [MB].

*Onthophagus (Palaeonthophagus) grossepunctatus* Reitter, 1905.

Localities. Donje Srijede at Presjeka, 28 May 2021, 1 ex. [MB], 1 ex. [AB]; Crnodoli at Nikšić, 27–28 May 2021, 1 ex. [MB], 1 ex. [ST]; Broćanac Nikšićki at Nikšić, 27 May 2021, 1 ex. [MB], 2 exx. [AB, ST]; Čevo at Cetynia, 27 May 2021, 2 exx. [AB, ST]; Vusanje at Gusinje, 21–22 May 2021, 1 ex. [AB].

*Onthophagus (Palaeonthophagus) lemur* (Fabricius, 1781).

Localities. Donje Srijede at Presjeka, 28 May 2021, 2 exx. [AB, ST]; Grahovo at Rožaje, 19–20 May 2021, 1 ex. [AB]; Riđani at Nikšić, 28 May 2021, 3 exx. [MB], 1 ex. [AB]; Broćanac Nikšićki at Nikšić, 27 May 2021, 1 ex. [MB], 4 exx. [AB, ST]; Donje Polje at Mrke, 22 May 2021, 1 ex. [AB]; Škala at Gusinje, 21 May 2021, 1 ex. [ST]; Vusanje at Gusinje, 21–22 May 2021, 2 exx. [AB, ST]; Žanjev Do at Njeguši, 25 May 2021, 4 exx. [AB, ST]; Dubovik at Cetynia, 25–27 May 2021, 1 ex. [ST]; Buljarica at Petrovac na Moru, 25 May 2021, 3 exx. [MB], 2 exx. [AB, ST].

*Onthophagus (Palaeonthophagus) medius* (Kugelann, 1792).

Localities. Trsa at Plužine, 29–30 May 2021, 2 exx. [MB], 8 exx. [AB, ST]; Donje Srijede at Presjeka, 28 May 2021, 8 exx. [AB, ST]; Broćanac Nikšićki at Nikšić, 27 May 2021, 8 exx. [AB, ST]; Plav, 20 May 2021, 3 exx. [MB], 3 exx. [AB, ST]; Žanjev Do at Njeguši, 25 May 2021, 3 exx. [AB, ST]; Dubovik at Cetynia, 25–27 May 2021, 4 exx. [AB, ST]; Bigova at Kotor, 15–17 July 2021, 2 exx. [DM].

*Onthophagus (Palaeonthophagus) opacicollis* Reitter, 1892.

Locality. Radigojno at Kolašin, 18 July 2021, 1 ex. [DM].

**Figure 7:**
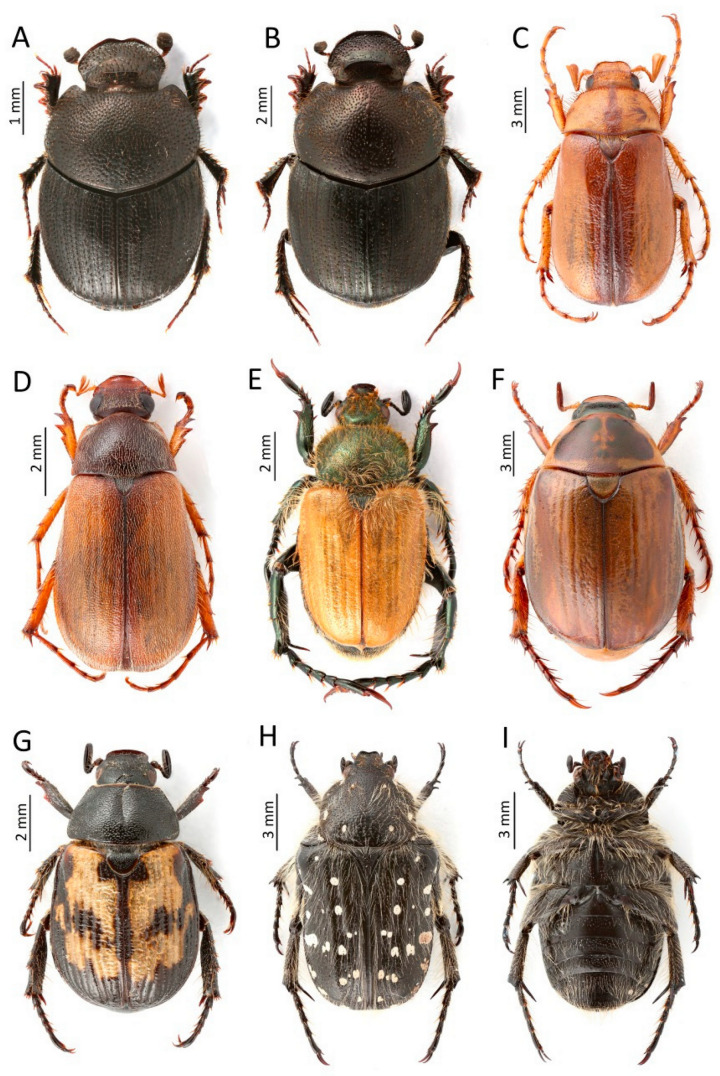
Rare and new species for the fauna of Montenegro were recorded in the course of this study: (**A**)—*Onthophagus ovatus, (**B**)—*O. panici*, (**C**)—*Rhizotrogus aestivus, (**D**)—*Triodontella dalmatica*, (**E**)—*Chaetopteroplia segetum, (**F**)—*Anomala matzenaueri*, (**G**)—*Exomala adriatica*, (**H**,**I**)—*Oxythyrea dulcis* (*—species new for the Montenegro fauna) (Photos: Łukasz Minkina).

*Onthophagus (Palaeonthophagus) ovatus (Linnaeus, 1767) (Figure 7A).

Localities. Crnodoli at Nikšić, 27–28 May 2021, 1 ex. [AB]; Riđani at Nikšić, 28 May 2021, 6 exx. [MB], 8 exx. [AB, ST]; Broćanac Nikšićki at Nikšić, 27 May 2021, 1 ex. [ST]; Čevo at Cetynia, 27 May 2021, 5 exx. [AB, ST]; Plav, 20 May 2021, 3 exx. [AB, ST].

Remarks. Reported from most European countries, including those around Montenegro (Albania, Bosnia Herzegovina, Croatia, Serbia) and also Azerbaijan, Georgia, Iran, Kazakhstan, Tajikistan and Turkey [34,35]. Imagines were caught on pastures at an elevation of about 600 to 1000 m above sea level, all from the excrement of sheep, cows and horses (Figure 3B–F).

*Onthophagus (Palaeonthophagus) panici* Petrovitz, 1964 (Figure 7B).

Localities. Trsa at Plužine, 29–30 May 2021, 1 ex. [AB]; Mala Crna Gora at Žabljak, 31 May 2021, 1 ex. [ST]; Stožina at Žabljak, 1–2 June 2021, 14 exx. [MB], 16 exx. [AB, ST], 1 ex. [TG]; Pošćenski Kraj at Žabljak, 31 May–1 June 2021, 23 exx. [MB], 21 exx. [AB, ST].

Remarks. Recorded from Balkan Peninsula: Albania, Bosnia Herzegovina, Greece, North Macedonia and Montenegro [24,35,36]. Imagines have been gathered on high mountain pastures with numerous tunnels dug by rodents (Figure 2A–C,E): most of them in ground-trapped using fresh sheep droppings for bait, the rest of them directly in sheep droppings, rarely in cows and horse’s faeces.

*Onthophagus (Palaeonthophagus) ruficapillus* Brullé, 1832.

Locality. Buljarica at Petrovac na Moru, 25 May 2021, 5 exx. [AB, ST].

*Onthophagus (Palaeonthophagus) sericatus* Reitter, 1892.

Localities. Trsa at Plužine, 29–30 May 2021, 1 ex. [MB]; Broćanac Nikšićki at Nikšić, 27 May 2021, 1 ex. [MB]; Dubovik at Cetynia, 25–27 May 2021, 1 ex. [MB]; Buljarica at Petrovac na Moru, 25 May 2021, 3 exx. [AB, ST].

*Onthophagus (Palaeonthophagus) vacca* (Linnaeus, 1767).

Localities. Bajovo Polje at Plužine, 9 May 2021, 2 exx. [AB, ST]; Radigojno at Kolašin, 18 July 2021, 3 exx. [DM]; Riđani at Nikšić, 28 May 2021, 2 exx. [AB, ST]; Čevo at Cetynia, 27 May 2021, 2 exx. [AB, ST]; Škala at Gusinje, 21 May 2021, 1 ex. [AB]; Buljarica at Petrovac na Moru, 25 May 2021, 1 ex. [ST].

*Onthophagus (Palaeonthophagus) verticicornis* (Laicharting, 1781).

Localities. Trsa at Plužine, 29–30 May 2021, 2 exx. [MB], 12 exx. [AB, ST]; Pošćenski Kraj at Žabljak, 31 May–1 June 2021, 1 ex. [MB], 1 ex. [AB]; Bajovo Polje at Plužine, 29 May 2021, 1 ex. [ST]; Donje Srijede at Presjeka, 28 May 2021, 4 exx. [MB], 10 exx. [AB, ST]; Grahovo at Rožaje, 19–20 May 2021, 1 ex. [MB], 6 exx. [AB, ST]; Broćanac Nikšićki at Nikšić, 27 May 2021, 20 exx. [AB, ST]; Čevo at Cetynia, 27 May 2021, 1 ex. [AB]; Škala at Gusinje, 21 May 2021, 1 ex. [MB], 6 exx. [AB, ST]; Vusanje at Gusinje, 21–22 May 2021, 4 exx. [MB], 3 exx. [AB, ST]; Žanjev Do at Njeguši, 25 May 2021, 4 exx. [MB], 3 exx. [AB, ST]; Dubovik at Cetynia, 25–27 May 2021, 5 exx. [MB], 4 exx. [AB, ST].

Tribe SCARABAEINI Latreille, 1802.

*Scarabaeus sacer* Linnaeus, 1758.

Locality. Bojana at Ulcinj, 23–24 May 2021, 1 ex. [MB], 2 exx. [AB, ST].

*Scarabaeus typhon* (Fischer von Waldheim, 1823).

Locality. Bojana at Ulcinj, 23–24 May 2021, 1 ex. [TG].

Tribe SISYPHINI Mulsant, 1842.

*Sisyphus schaefferi schaefferi* (Linnaeus, 1758).

Localities. Donje Srijede at Presjeka, 28 May 2021, 1 ex. [MB], 2 exx. [AB, ST]; Broćanac Nikšićki at Nikšić, 27 May 2021, 3 exx. [MB], 7 exx. [AB, ST]; Čevo at Cetynia, 27 May 2021, 1 ex. [TG]; Dubovik at Cetynia, 25–27 May 2021, 1 ex. [MB], 2 exx. [AB, ST].

Subfamily MELOLONTHINAE Leach, 1819.

Tribe HOPLIINI Latreille, 1829.

*Hoplia argentea* (Poda von Neuhaus, 1761).

Localities. Muskovica Rijeka at Kolašin, 20 July 2021, 1 ex. [DM]; Žanjev Do at Njeguši, 25 May 2021, 1 ex. [MB], 1 ex. [TG]; Dubovik at Cetynia, 25–27 May 2021, 1 ex. [MB], 1 ex. [AB].

*Hoplia hungarica* Burmeister, 1844.

Locality. Bojana at Ulcinj, 23–24 May 2021, 8 exx. [MB], 4 exx. [AB, ST], 12 exx. [TG].

Remarks. Species inhabiting Europe (Albania, Bosnia Herzegovina, Belarus, Croatia, Czech Republic, France, Germany, Hungary, North Macedonia, Montenegro, Poland, Serbia, Slovakia, Slovenia, Spain and Ukraine) [37]. Widely distributed but rarely observed. This species was observed between 10 a.m. and 4 p.m. Several living and many dead individuals were found on the sand surface among tall reeds adjacent to the seashore dunes.

Tribe MELOLONTHINI Leach, 1819.

*Melolontha pectoralis pectoralis* Megerle von Mühlfeld, 1812.

Localities. Trsa at Plužine, 29–30 May 2021, 1 ex. [TG]; Stožina at Žabljak, 1–2 June 2021, 1 ex. [MB], 1 ex. [AB], 1 ex. [TG]; Dubovik at Cetynia, 25–27 May 2021, 11 exx. [MB], 22 exx. [AB, ST], 17 exx. [TG].

Tribe RHIZOTROGINI Burmeister, 1855.

*Amphimallon assimile* (Herbst, 1790).

Locality. Muskovica Rijeka at Kolašin, 20 July 2021, 1 ex. [DM].

*Amphimallon solstitiale simplicissimum* (J. Müller, 1902).

Localities. Meljine at Herceg Novi, 28 June–2 July 2019, 4 exx. [AB], det. A. Matusiak; Bigova at Kotor, 15–17 July 2021, 1 ex. [DM].

Remarks. The subspecies is known only from two countries: Albania [38] and Montenegro (Herceg Novi, Bar [7], Budva, Buljarica, Sutomore, Virpazar [39]). Its occurrence is limited to the Adriatic coast from Lake Skadar to the Bay of Kotar. The mountains Rumija, Lovćen and Orjen separate this narrow (1.5 to 6 km wide) lowland coastal strip from the rest of the country. The highest rainfall is in autumn and winter. On the slopes facing the sea, it exceeds 4000 mm per year. Probably the specific climate conditions have caused the separation of these subspecies. Males flew in the same place as *Aplidia transversa* (Fabricius, 1801) only for a short time, between 8 p.m. and 9 p.m.

*Amphimallon solstitiale solstitiale* (Linnaeus, 1758).

Locality. Stožina at Žabljak, 1–2 June 2021, 3 exx. [MB].

*Aplidia transversa transversa* (Fabricius, 1801).

Localities. Meljine at Herceg Novi, 28 June–2 July 2019, 14 exx. [AB]; Bigova at Kotor, 15–17 July 2021, 2 exx. [DM].

*Rhizotrogus aestivus (A.G. Olivier, 1789) (Figure 7C).

Localities. Trsa at Plužine, 29–30 May 2021, 1 ex. [MB]; Crnodoli at Nikšić, 27–28 May 2021, 1 ex. [MB], 1 ex. [AB], 2 exx. [TG]; Dubovik at Cetynia, 25–27 May 2021, 11 exx. [MB], 16 exx. [AB, ST], 11 exx. [TG].

Remarks. The species were reported from most European countries (including Montenegro’s neighbours: Albania, Bosnia Herzegovina, Croatia, Serbia), as well as Iran, Kazakhstan, Syria and Turkey [38]. Specimens of this species were caught at dusk during their flight in an insect net and at night on an insect beating sheet, and in an insect sweep net. A copulating couple of this species has been spotted on low-lying oak leaves in the oak forests and bushy pastures (Figure 2A, Figure 3B and Figure 4E).

Tribe SERICINI Kirby, 1837.

*Maladera holosericea* (Scopoli, 1772).

Localities. Crnodoli at Nikšić, 27–28 May 2021, 21 exx. [MB], 37 exx. [AB, ST], 23 exx. [TG]; Dubovik at Cetynia, 25–27 May 2021, 10 exx. [MB], 4 exx. [AB, ST].

*Omaloplia illyrica* (Baraud, 1965).

Locality. Pošćenski Kraj at Žabljak, 31 May–1 June 2021, 1 ex. [MB], det. E. Rößner.

*Omaloplia ruricola ruricola* (Fabricius, 1775).

Localities. Dobrilovina at Mojkovac, 19 July 2021, 2 exx. [DM], det. E. Rößner; Lipovska Bistrica at Kolašin, 20 July 2021, 1 ex. [DM].

*Triodontella dalmatica* (Baraud, 1962) (Figure 7D).

Localities. Žanjev Do at Njeguši, 25 May 2021, 1 ex. [MB], 2 exx. [AB, ST], 1 ex. [TG]; Dubovik at Cetynia, 25–27 May 2021, 3 exx. [MB], 6 exx. [TG].

Remarks. A species found in south-eastern Europe (Albania, Bosnia Herzegovina, Bulgaria, Croatia, Greece, Kosovo, North Macedonia, Montenegro, Romania, Serbia and the European part of Turkey) [40,41]. All specimens were caught at dusk or night in beech and oak forests (Figure 4C,E). Most of them were caught on an insect beating sheet and in an insect sweep net. A copulating couple of this species was spotted on low-lying beech leaves.

Subfamily RUTELINAE W.S. Macleay, 1819.

Tribe ANOMALINI Streubel, 1839.

Subtribe ANISOPLIINA Burmeister, 1844.

*Anisoplia flavipennis* Brullé, 1832.

Locality. Buljarica at Petrovac na Moru, 25 May 2021, 2 exx. [MB], 1 ex. [AB], 9 exx. [TG], det. A. Bezděk.

*Chaetopteroplia segetum straminea (Brullé, 1832) (Figure 7E).

Localities. Doni Štoj at Ulcinj (pasture), 24 May 2021, 2 exx. [TG]; Sveti Nikola at Ulcinj, 23 May 2021, 13 exx. [MB], 20 exx. [AB, ST], det. E. Rößner.

Remarks. One of the eleven described subspecies of the widely distributed species *Chaetopteroplia segetum* (Herbst, 1783). Species inhabiting Europe, Kazakhstan, Turkey and Turkmenistan. Subspecies *Ch. segetum straminea* inhabits Albania, Bulgaria, North Macedonia and Greece [42]. Individuals of this subspecies were observed in the afternoon on the ears of grass on two pastures and in their immediate vicinity.

Subtribe ANOMALINA Streubel, 1839.

*Anomala dubia dubia* (Scopoli, 1763).

Locality. Bojana at Ulcinj, 23–24 May 2021, 7 exx. [MB], 11 exx. [AB, ST], 3 exx. [TG].

*Anomala matzenaueri* Reitter, 1918 (Figure 7F).

Locality. Bojana at Ulcinj, 23–24 May 2021, 8 exx. [MB], 11 exx. [AB, ST], 4 exx. [TG].

Remarks. The species is known only from two countries: Albania and Montenegro [17,42,43]. Single individuals of this species were found sitting or buried in the sand, but most individuals were caught in insect nets. They flew low for a short time (between 8 p.m. and 9 p.m.) at dusk and early night among tall reeds adjacent to the seashore dunes (Figure 4G).

*Anomala vitis* (Fabricius, 1775).

Locality. Bojana at Ulcinj, 23–24 May 2021, 1 ex. [AB].

*Exomala adriatica* (Petrovitz, 1968) (Figure 7G).

Locality. Bojana at Ulcinj, 23–24 May 2021, 1 ex. [MB], 3 exx. [AB, ST], 2 exx. [TG].

Remarks. Species only reported from three countries: Albania, Greece and Montenegro [17,42]. All specimens of this species were collected in a holiday resort located by the sea (Figure 4H). Most of them were found on the sidewalk and single ones in the nearby grass.

Subfamily DYNASTINAE W.S. Macleay, 1819.

Tribe ORYCTINI Mulsant, 1842.

*Oryctes nasicornis kuntzeni* Minck, 1914.

Localities. Meljine at Herceg Novi, 28 June–2 July 2019, 1 ex. [AB]; Bigova at Kotor, 15–17 July 2021, 1 ex. [DM].

Tribe PENTODONTINI Mulsant, 1842.

*Pentodon idiota idiota* (Herbst, 1789).

Localities. Bigova at Kotor, 15–17 July 2021, 1 ex. [DM]; Doni Štoj at Ulcinj (dunes), 24 May 2021, 1 ex. [MB], 1 ex. [AB]; Bojana at Ulcinj, 23–24 May 2021, 4 exx. [AB, ST], 1 ex. [TG].

*Phyllognathus excavatus* (Forster, 1771).

Locality. Bigova at Kotor, 15–17 July 2021, 2 exx. [DM].

Subfamily CETONIINAE Leach, 1819.

Tribe CETONIINI Leach, 1819.

Subtribe CETONIINA Leach, 1819.

*Cetonia aurata aurata* (Linnaeus, 1758).

Localities. Bajovo Polje at Plužine, 29 May 2021, 1 ex. [MB], 1 ex. [AB]; Bistrica at Mojkovac, 23 July 2021, 2 exx. [DM]; Brskovo at Mojkovac, 18 July 2021, 2 exx. [DM]; Donje Srijede at Presjeka, 28 May 2021, 2 exx. [MB]; Muskovica Rijeka at Kolašin, 20 July 2021, 1 ex. [DM]; Čevo at Cetynia, 27 May 2021, 4 exx. [AB, ST]; Donje Polje at Mrke, 22 May 2021, 1 ex. [ST]; Vusanje at Gusinje, 21–22 May 2021, 1 ex. [AB]; Žanjev Do at Njeguši, 25 May 2021, 1 ex. [AB]; Dubovik at Cetynia, 25–27 May 2021, 1 ex. [MB]; Bigova at Kotor, 15–17 July 2021, 1 ex. [DM]; Buljarica at Petrovac na Moru, 25 May 2021, 3 exx. [AB, ST]; Bojana at Ulcinj, 23–24 May 2021, 2 exx. [MB].

*Protaetia (Cetonischema) speciosissima* (Scopoli, 1786).

Locality. Crnodoli at Nikšić, 27–28 May 2021, 1 ex. (dead) [TG].

*Protaetia (Eupotosia) affinis affinis* (Andersch, 1797).

Localities. Meljine at Herceg Novi, 28 June–2 July 2019, 1 ex. [AB]; Žanjev Do at Njeguši, 25 May 2021, 1 ex. [AB].

*Protaetia (Potosia) angustata angustata* (Germar, 1817).

Localities. Meljine at Herceg Novi, 28 June–2 July 2019, 3 exx. [AB]; Bigova at Kotor, 15–17 July 2021, 3 exx. [DM]; Buljarica at Petrovac na Moru, 25 May 2021, 1 ex. [AB].

*Protaetia (Potosia) cuprea obscura* (Andersch, 1797).

Localities. Dubovik at Cetynia, 25–27 May 2021, 1 ex. (dead) [TG]; Bojana at Ulcinj, 23–24 May 2021, 1 ex. (dead) [ST].

*Tropinota (Epicometis) hirta hirta* (Poda von Neuhaus, 1761).

Localities. Bajovo Polje at Plužine, 29 May 2021, 1 ex. [AB]; Čevo at Cetynia, 27 May 2021, 2 exx. [AB, ST]; Donje Polje at Mrke, 22 May 2021, 1 ex. [TG]; Škala at Gusinje, 21 May 2021, 3 exx. [MB], 2 exx. [AB, ST]; Žanjev Do at Njeguši, 25 May 2021, 1 ex. [ST]; Dubovik at Cetynia, 25–27 May 2021, 1 ex. [MB], 3 exx. [AB, ST], 2 exx. [TG]; Doni Štoj at Ulcinj (dunes), 24 May 2021, 1 ex. [AB]; Bojana at Ulcinj, 23–24 May 2021, 1 ex. [MB], 3 exx. [AB, ST], 1 ex. [TG].

*Tropinota (Tropinota) squalida squalida* (Scopoli, 1763).

Localities. Dubovik at Cetynia, 25–27 May 2021, 2 exx. [MB], 1 ex. [AB]; Buljarica at Petrovac na Moru, 25 May 2021, 1 ex. [TG]; Doni Štoj at Ulcinj (pasture), 24 May 2021, 1 ex. [TG].

Subtribe LEUCOCCELNA Kraatz, 1882.

*Oxythyrea dulcis* Reitter, 1899 (Figure 7H,I).

Locality. Bojana at Ulcinj, 23–24 May 2021, 8 exx. [MB], 10 exx. [AB, ST], 3 exx. [TG].

Remarks. Species were only reported from three countries: Greece, Montenegro and Turkey [44,45,46]. Individuals of this species were observed in the morning between 9 a.m. and 11 a.m. Imagines sat on the flowers of plants growing on coastal dunes (Figure 4G).

*Oxythyrea funesta* (Poda von Neuhaus, 1761).

Localities. Dobrilovina at Mojkovac, 19 July 2021, 2 exx. [DM]; Bistrica at Mojkovac, 23 July 2021, 4 exx. [DM]; Brskovo at Mojkovac, 18 July 2021, 4 exx. [DM]; Lipovska Bistrica at Kolašin, 20 July 2021, 2 exx. [DM]; Donje Polje at Mrke, 22 May 2021, 2 exx. [AB, ST]; Meljine at Herceg Novi, 28 June–2 July 2019, 1 ex. [AB]; Žanjev Do at Njeguši, 25 May 2021, 3 exx. [AB, ST]; Dubovik at Cetynia, 25–27 May 2021, 7 exx. [AB, ST], 1 ex. [TG]; Bigova at Kotor, 15–17 July 2021, 2 exx. [DM]; Buljarica at Petrovac na Moru, 25 May 2021, 1 ex. [MB], 4 exx. [AB, ST]; Sveti Nikola at Ulcinj, 23 May 2021, 3 exx. [AB, ST]; Bojana at Ulcinj, 23–24 May 2021, 5 exx. [AB, ST].

Tribe TRICHIINI Fleming, 1821.

*Gnorimus nobilis nobilis* (Linnaeus, 1758).

Localities. Lipovska Bistrica at Kolašin, 20 July 2021, 1 ex. [DM]; Muskovica Rijeka at Kolašin, 20 July 2021, 2 exx. [DM].

*Gnorimus variabilis* (Linnaeus, 1758).

Locality. Muskovica Rijeka at Kolašin, 20 July 2021, 1 ex. [DM].

*Trichius sexualis* Bedel, 1906.

Localities. Bistrica at Mojkovac, 23 July 2021, 4 exx. [DM]; Brskovo at Mojkovac, 18 July 2021, 1 ex. [DM]; Lipovska Bistrica at Kolašin, 1180 m a.s.l., 20 July 2021, 2 exx. [DM]; Donje Polje at Mrke, 22 May 2021, 1 ex. [AB]; Buljarica at Petrovac na Moru, 25 May 2021, 1 ex. [TG].

Tribe VALGINI Mulsant 1842.

*Valgus hemipterus hemipterus* (Linnaeus, 1758).

Localities. Donje Polje at Mrke, 22 May 2021, 1 ex. [MB], 2 exx. [AB, ST]; Žanjev Do at Njeguši, 25 May 2021, 4 exx. [AB, ST]; Dubovik at Cetynia, 25–27 May 2021, 7 exx. [AB, ST]; Buljarica at Petrovac na Moru, 25 May 2021, 11 exx. [AB, ST]; Bojana at Ulcinj, 23–24 May 2021, 1 ex. [ST].

## 4. Discussion

The “Catalogue of Palaearctic Coleoptera” [1] contains 163 scarabaeoid species from Montenegro. The occurrence of three species in Montenegro—*Jekelius punctulatus* (Jekel, 1866) [10,47], *Trichiorhyssemus dalmatinus* Petrovitz, 1967 and *Amphimallon burmeisteri* Brenske, 1886 [10] has been omitted in the second edition of the “Catalogue of Palaearctic Coleoptera” [1]. Král et al. [26] supplemented the list of Montenegrin scarabaeoids with two more species: *Erytus aequalis* and *Euorodalus tersus*. Thus, up to 168 species of Scarabaeoidea have been identified in Montenegro.

The 28 days of faunistic study resulted in confirming the occurrence in Montenegro of 54.2% of the scarabaeoid species hitherto known from this country and added 16 species that had not been previously recorded: *Odonteus armiger*, *Trox sabulosus*, *Ochodaeus integriceps*, *Agrilinus convexus*, *Melinopterus reyi*, *M. sphacelatus*, *Phalacronothus biguttatus*, *Trichonotulus scrofa*, *Psammodius nocturnus*, *Platytomus tibialis*, *Pleurophorus mediterranicus*, *P. pannonicus*, *Rhyssemus berytensis*, *Onthophagus ovatus*, *Rhizotrogus aestivus* and *Chaetopteroplia segetum*. Thus, the number of currently known scarabaeoid species in Montenegro has increased to 184.

Six species and three subspecies that are typical for the region of Balkan Peninsula were also found: *Trypocopris alpinus balcanicola*, *Onthophagus panici*, *Amphimallon solstitiale simplicissimum*, *Omaloplia illyrica*, *Triodontella dalmatica*, *Chaetopteroplia segetum straminea*, *Anomala matzenaueri*, *Exomala adriatica* and *Oxythyrea dulcis*. These species were observed at single localities.

The diversity of scarabaeoid fauna on the Balkan Peninsula suggests that this number may be increased further. Kulundžić et al. [48] estimated that more than 400 scarabaeoid species occur in Croatia, while Guéorguiev et al. [49] estimated the number of scarabaeoid species in Bulgaria at 335–345. As regards the adjoining countries, 240 scarabaeoid species are currently known from Croatia, 214 from Kosovo and Serbia, 204 from Bosnia Herzegovina [1] and 201 from Albania [17]. In the first edition of the „Catalogue of Palaearctic Coleoptera" [11], there are 156 scarabaeoid species mentioned from Albania. Ten years later, its second edition [1] already listed 181 species, and 3 years later, the number of species reached 201 [17].

The richness of the scarabaeoid fauna of Montenegro and the geography of this country clearly indicates the possibility of finding further species of this superfamily, in particular near the borders with neighbouring countries where numerous other taxa have been recorded. So far, from the territory of Montenegro, the following species have never been recorded: *Trox scaber* (Linnaeus, 1767) [20], *Amidorus cribrarius* (Brullé, 1832), *Eupleurus subterraneus* (Linnaeus, 1758), *Liothorax plagiatus* (Linnaeus, 1767), *Loraphodius suarius* (Faldermann, 1835) [25], *Psammodius laevipennis* Costa, 1844 [32], *Hoplia brunnipes* Bonelli, 1812, *H. dilutipes* Reitter, 1890 [37], *Serica brunnea* (Linnaeus, 1758) [39], *Anisoplia agricola* (Poda von Neuhaus, 1761), *A. lata* Erichson, 1847, *Mimela aurata* (Fabricius, 1801), *Phyllopertha horticola* (Linnaeus, 1758) [42], *Protaetia marmorata* (Fabricius, 1792), *P. fieberi* (Kraatz, 1880) and *Trichius fasciatus* (Linnaeus, 1758) [46]. All these species are known from all neighbouring countries such as Albania, Croatia, Bosnia Herzegovina and Serbia. Some of them are common species in these countries, and there are suitable habitats available for them in Montenegro.

Over the last years, ten new species of scarabaeoid beetles for Montenegro were recorded. Some years ago, Ziani et al. [24] reported eight species of scarabaeoid beetles from Montenegro for the first time: *Dorcus parallelipipedus*, *Platycerus caraboides* (Linnaeus, 1758), *Chilothorax conspurcatus* (Linnaeus, 1758), *Brindalus porcicollis* (Illiger, 1803), *Onthophagus opacicollis*, *O. sericatus*, *Polyphylla boryi* (Brullé, 1832) and *Firminus lautiusculus* (Schaufuss, 1864). Last year, Král et al. [26] reported the discovery of two other species in this country, i.e., *Erytus aequalis* and *Euorodalus tersus*.

Moreover, rare species of scarabaeoid beetles are reported from nearby countries, e.g., *Trox perrisii* Fairmaire, 1868 from Croatia [24,50], Bulgaria [30] and Greece [21] or the Albanian endemic *Ahermodontus bischoffi* Všetečka, 1939 [51]. At least the first of the listed species may occur in Montenegro.

## 5. Conclusions

Within less than a month of fieldwork, the discovery of 16 species new to Montenegro also points to the insufficient state of knowledge of its scarabaeoid fauna. Hence, there is a need to continue and intensify faunistic studies, especially in the hitherto less explored areas. The diversity of the scarabaeoid fauna of Montenegro is certainly greater. Among the 107 species of scarabaeoid beetles observed during our field studies, 63 of them are coprophagous. Montenegro is a European country where, due to the traditional form of grazing, dung beetle communities are characterised by a considerable richness of species and the presence of rare species. Three important aspects for the conservation of dung beetle diversity emerge from this work: saving the traditional form of domestic animal grazing, law for the protection of well-preserved pastures and active protection in order to ensure adequate living conditions for rare species. The third of the above-mentioned aspects is also important for preserving beetle communities inhabiting the Montenegrin coastal dunes. Recommended protection of dung beetles should be based on maintenance and introduction of small-size herds of goats and sheep, single cows and horses on mountain pastures. Then, the protection of psammophilous scarabaeoid beetles should include the removal of garbage from coastal dunes and the prevention of these habitats from being overgrown by shrubs and trees. Equally important is the possibility of conducting further research on the structure of these communities. The importance of the traditional grazing system and the importance of long-term research were clearly demonstrated by Tretler et al. [52]. The cited authors found that as much as 51% of Sardinian coprophagic scarabaeoid beetles occur on the small Asinara island (only 51.9 km^2^).

## Data Availability

Evidence material (the specimens presented) has been deposited in the entomological collections of the Department of Forest Protection of the Warsaw University of Life Sciences and in the private collections of the authors.
